# These Aren’t The Droids You Are Looking for: Promises and Challenges for the Intersection of Affective Science and Robotics/AI

**DOI:** 10.1007/s42761-023-00211-3

**Published:** 2023-08-18

**Authors:** Arvid Kappas, Jonathan Gratch

**Affiliations:** 1 Constructor University, Campus Ring 1, 28759 Bremen, Germany; 2 Institute for Creative Technologies, University of Southern California, Los Angeles, CA USA

**Keywords:** Robotics, Affective computing, Human robot interaction, Artificial intelligence

## Abstract

AI research focused on interactions with humans, particularly in the form of robots or virtual agents, has expanded in the last two decades to include concepts related to affective processes. Affective computing is an emerging field that deals with issues such as how the diagnosis of affective states of users can be used to improve such interactions, also with a view to demonstrate affective behavior towards the user. This type of research often is based on two beliefs: (1) artificial emotional intelligence will improve human computer interaction (or more specifically human robot interaction), and (2) we understand the role of affective behavior in human interaction sufficiently to tell artificial systems what to do. However, within affective science the focus of research is often to test a particular assumption, such as “smiles affect liking.” Such focus does not provide the information necessary to synthesize affective behavior in long dynamic and real-time interactions. In consequence, theories do not play a large role in the development of artificial affective systems by engineers, but self-learning systems develop their behavior out of large corpora of recorded interactions. The status quo is characterized by measurement issues, theoretical lacunae regarding prevalence and functions of affective behavior in interaction, and underpowered studies that cannot provide the solid empirical foundation for further theoretical developments. This contribution will highlight some of these challenges and point towards next steps to create a rapprochement between engineers and affective scientists with a view to improving theory and solid applications.

About 100 years ago, the term *robot* was introduced in the context of Karel Čapek’s play R.U.R.: Rossum’s Universal Robots (1920). While the idea of artificial humans had been a topic of literature and film before (e.g., the Golem, Frankenstein, Metropolis), R.U.R. was a turning point and provided a label and a concept that would quickly spread internationally. Within a few decades robots and other humanoid artificial creatures would be common in science fiction stories, film and later television and video games. One of the interesting aspects of these artificial creatures was that typically, they would be presented as smart, possessing (artificial) intelligence, but being cold, distant, and unemotional. In fact, emotions seemed to be the missing element in truly obtaining humanity, such as the character *Lt. Cmdr. Data* in the Star Trek universe (Kakoudaki, 2015). Indeed, several studies suggest that emotion has become an even more crucial aspect of human identity in response to the inexorable rise in machine intelligence (e.g., Cha et al., 2020; Kaplan, 2004; Stein & Ohler, 2017).

About 20 years ago, Rosalind Picard (1997) introduced the concept *affective computing* and ever since, a broad and heterogeneous research program linking AI and affective science has been growing rapidly. While research in this context existed before (see also Picard, 2015), it did not present a cohesive body of activities and was not perceived as such. After the turn of the millennium, in a relatively short time, societies, conferences, and journals centered around the new concept appeared and grew at a rapid pace. The IEEE flagship journal *IEEE Transactions on Affective Computing*, founded in 2010, reached soon a higher impact factor than any canonical journal on emotions/affective science (13.99 at the time of writing). This remarkable expansion correlates with the current growth of artificial intelligence in the guise of machine learning and data analytic approaches that are transformative in many disciplines and applied areas on the one hand and the rise of *affectivism* on the other (Dukes et al., 2021).

The present contribution will take stock of the state of affective science in affective computing and social robotics. We will highlight challenges to implementing affect in machines and discuss the potential benefits for researchers in the field of affective science in the coming years to connect with researchers involved in affective computing, AI, and social robotics.

## Motivations for Development of Affective Computing

Many researchers in affective computing are interested in developing systems that are supposed to gain usability in the widest sense in the interaction of humans and artificial systems. Benefits are proposed for physically embodied systems, such as robots (HRI: human robot interaction), or virtual entities, such as virtual agents or chatbots. Designers and researchers hope that by diagnosing the state of users or interactants, such systems can alter their behavior or convey simulated emotions to better fit the situation, or the needs of the user. Service providers could identify angry customers and respond with empathy or concern, or at least transition them to a human representative (e.g., Waelbers et al., 2022). Home devices like Alexa might target ads to when a customer is emotionally predisposed to purchase (Li et al., 2017). Automated tutors might detect student frustration and provide encouragement or adjust instruction accordingly (Malekzadeh et al., 2015). Because of the implications of being able to diagnose user states and develop responsive systems, there is a considerable business case. Studies from the year 2022 estimate the global affective computing market by 2026 between 182 and 255 billion US$ (Reports and data, 2022). Arguably, there is no aspect of affective science research that surpasses the current market interest of affective computing. It is all the more relevant that the connections between emotion researchers from the behavioral-, social-, and neurosciences and much of the affective computing enterprise are comparatively weak. It should also be noted, that particularly in the context where information on affective states is being used to sell products, concepts, or services, there are considerable ethical issues. These concerns are being discussed by experts at conferences and in the literature, as well as by the media in public discourse. This is an ongoing discussion that we can only mention and not pursue in this overview.

In contrast, a smaller group of researchers is interested in developing artificial agents that represent an internal affective state, in this case, the idea is that the behavior of such agents will be determined by the co-action of cognition, affect, and motivation (e.g., Lim & Okuno, 2015). Attempts to create feeling machines are not frequent and have not yet been very successful though there is recent excitement that “foundation” models like GPT-3 may have spontaneously acquired socio-emotional abilities (Kosinski, 2023)—a claim that must be taken with healthy skepticism (see Ullman, 2023). As such artificial actions begin to collaborate within human individuals and groups, an important goal might be to understand the social role of artificial actors, such as whether they are part of social groups and are subject to social cognitive processes beyond the individual interaction (Vanman & Kappas, 2019).

On the side of affective science, there is arguably an interest in using artificial systems as methodological tools to advance emotion theory. Robots and virtual characters have been argued to hold advantages over human confederates by allowing highly controlled experimental manipulations of expressive behavior while avoiding experimenter effects (e.g., Pan & Hamilton, 2018). For example, virtual partner expressions have been used to examine how cooperation is shaped by different patterns of the partner’s expressed emotion in social decision-making tasks (de Melo et al., 2014) or to uncover the neural correlates of self- versus other-directed emotional expressions (Schilbach et al., 2006). Of course, expressed emotions are an important aspect of psychological processes and automated methods offer the possibility of measuring these signals at much larger scale than possible with trained human annotators. For example, automatically sentiment analysis was applied to the Facebook feeds of half a million people to study emotional contagion in social networks (Kramer et al., 2014) and to 600 million tweets to assess theories of what makes sporting events exciting (Lucas et al., 2017). When it comes to facial expressions, full coding 1 min of dyadic interaction in Ekman and Friesen’s Facial Action Coding System (1978, see also below) is estimated to take 200 min of coding, limiting researchers to short interactions or small numbers of participants. In contrast, a recent studying applied automatic FACS coding to examine the facial dynamics of 750,000 participants (McDuff et al., 2017).

Lastly, there is an interest, particularly, in psychology and in communication studies, to analyze how people perceive and interact with artificial entities, given their increasing presence in society. Here, topics range from the influence of embodiment, behaviors, or culture. This work faces two challenges: (1) because of the complexity of having people interact with real robots, there is a chronic issue of statistically underpowered studies, be they in the lab or in the wild (see, e.g., a meta-analysis of studies on children’s trust in robots by Stower et al., 2021). (2) Even if there was not an issue of statistical power, it is not clear how valid the findings could be. As still few people have social robots regularly in their immediate surroundings, there is a particular interest in how stable findings are that might be linked to initial perceptions that might be driven by novelty. An exception are virtual intelligent assistants, such as Alexa, Siri, Google Assistant, or Cortana, as these have found their way into millions of homes. However, most of these systems do not embody emotional intelligence. Yet.

## Diagnosis of Affective States in the Context of Affective Computing

Much of the initial work in diagnosing affective states is explicitly or implicitly framed in terms of a readout hypothesis (e.g., Buck, 1994), where expressive behavior, particularly expressive behavior in the face, is seen as a direct indicator of the underlying affective state. Of prominence here are frameworks proposing a small number of discrete emotional states with clear and well-defined patterns, such as the work of Paul Ekman and his colleagues. Proprietary or open systems map the presence of particular facial actions to a label of an emotion. The systems can be based on an analysis of a group of preselected faces, or objective action units (AUs), as defined by Ekman and Friesen in their Facial Action Coding System (1978). In this logic, if someone displays a smile, as defined by the action of Zygomaticus Major (AU12) in the lower face and the action of Orbicularis Oculi (AU6) corresponding to crow´s feet wrinkles around the eyes, the presence of Happiness is diagnosed. This approach is highly problematic as the relationship between expressive behavior and the presence of the subjective experience of an emotion and/or changes in physiological activation consistent with affective states is far from a one-to-one relationship (e.g., Krumhuber & Kappas, 2022). Thus, even if the measurement of facial activation would be reliable, it is not possible to determine reliably affective state in any specific moment based on expressive behavior alone.

**Fig. 1:**
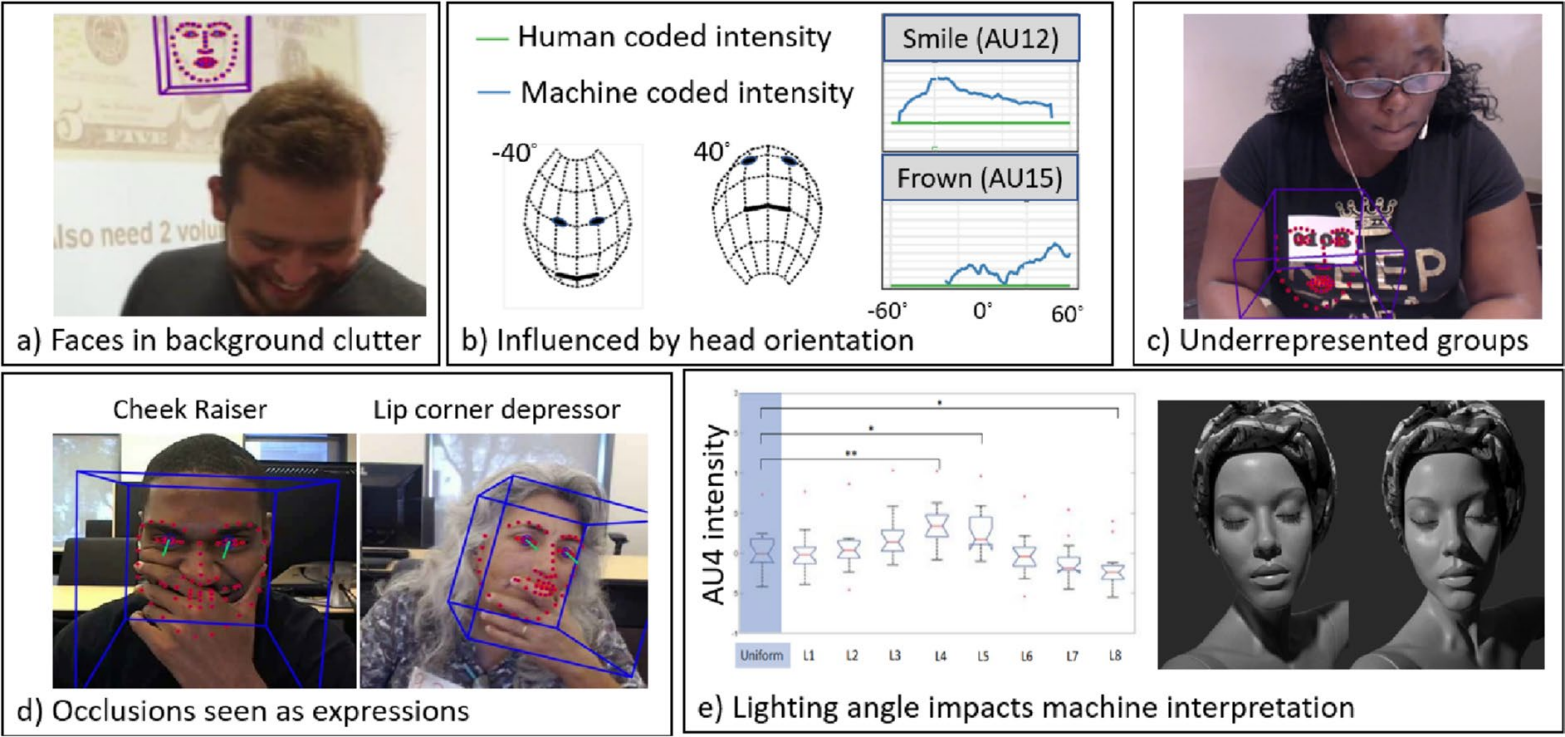
Expression recognition errors can arise from several factors

Adding to the conceptual problem of equating specific expressive patterns with the presence of a well-defined affective state, there is the issue that automated measurement can introduce systematic error or bias. Figure 1 illustrates common errors that arise in automatic FACS coding including (a) finding faces in background clutter, (b) report different action units as a function of head orientation (see Kappas et al., 1994), (c) failing to recognize minority faces (Xu et al., 2020), (d) ignoring the influence of occlusions (Zhang et al., 2018), and (e) being very sensitive to lighting conditions (Stratou et al., 2012), though each of these biases are under active investigation and some improvements have already made their way into commercial systems (Raji & Buolamwini, 2019).

## Creating Emotional Expressions

Emotional expressions may serve key functions in human social interactions and affective computing research is actively focused on creating expressive machines that, for example, build emotional connections with customers (van Doorn et al., 2017), motivate frustrated students (McQuiggan et al., 2008), or lower patient anxiety in clinical interviews (Lucas et al., 2014). Such systems must address *when* an expression should be generated (e.g., under what circumstances should a robot smile or look concerned) and how to render that expression into human-perceivable cues such as facial expressions, vocal prosody and behavior. In answering these questions, affective computing researchers typically ignore affective science findings and rely on stereotypes or their own intuitions. For example, Darwin (1872) suggested that surprise is accompanied by a raising of the eyebrows—a notion that is shared by researchers in the Darwinian tradition, such as Ekman and his colleagues (here operationalized as Action Units 1 + 2). Yet, Reisenzein et al. (2019) In a thorough sequence of empirical studies could demonstrate that this facial movement only rarely occurs in surprising situations. Similarly, Krumhuber and Kappas (2022) challenge the presence of the “enjoyment smile” as a reliable correlate of enjoyment. There is clear evidence that such smiles (Action Units 12 + 6; lower face smile and wrinkles around the eyes; also referred to as “Duchenne Smiles”) do not reliably occur with enjoyment, or that enjoyment does not reliably occurs when people show such smiles.

In deciding what expression to produce, designers often follow Buck’s (1994) readout hypothesis. In other words, they try to develop models of what a person might likely feel in a particular situation and link expressive behaviors to that model (e.g., Dias & Paiva, 2005). Rarely are expressions seen as pragmatic communicative acts, in a specific context, as suggested by several theories (e.g., Barrett et al., 2019; Fridlund, 1991, see also Krumhuber & Kappas, 2022). In terms of how these expressions are manifest, research is increasingly favoring generative machine learning approaches. The idea here is to learn to recognize facial expressions from human data and then “invert” these models to synthesize behavior (e.g., Hajarolasvadi et al., 2020). A concern with such approaches is that it is notoriously difficult to extract what a machine learning approach has actually learned, thus making it hard to connect such models to existing descriptive frameworks for characterizing expressions, such as Ekman and Friesen’s FACS, though others might argue this is also an advantage.

## Communication and Interaction

Synthesizing behavior for interactions with users involves several different aspects. There is a long history of developing conversational systems, going back at least to Weizenbaum’s Eliza (1966), a simple chatting system simulating a psychotherapist. Since then, there has been a constant development of systems that are able to hold a conversation in text in specific areas, such as education (e.g., Wollny et al., 2021) or health care (e.g., Parmar et al., 2022). However, if systems are to be embodied, a multi-modal synthesis approach is needed that involves not only *what* is being said, but *how* it is said, in the sense of involving paralinguistic cues and nonverbal behavior in general. Multimodal synthesis of behavior is hampered by the many degrees of freedom of behavior on the one hand, and the lack of theories that cover all different behavioral dimensions. Furthermore, there are many technical challenges with issues, such as synthesizing speech and mouth movements in a synchronous fashion in real time.

Clearly emotional expressions are part and parcel of behavior shown in interactions, but what and when they are shown is typically not covered in emotion theories. Being able to create a working system that shows expressions that relate to affective states, involves a joint effort of multiple disciplines, that involve psychology, communications, possibly linguistics, sociology, ethology, and more. Alternatively, one simply records many interactions and AI can produce behavior without recourse to any theory—is this really what we want? We know that generative processes depend on data being fed. Theories help to identify conditions and contexts that should be included in sampling the data for machine learning, as it is simply not viable to sample all of human behavior in all contexts with all of the facets that might play a role in the cohesion of affective components.

## Discussion

There is no doubt that affective computing is a growth industry in computer science and engineering and in some corners of affective science. However, while there is already huge interest on the business side, there are various issues that provide challenges on the scientific backbone of such developments. These lacunae are areas that are looking for serious investment in research activity.

We do not know the actual relationship of visible/audible affective behavior and underlying subjective experience and physiological activation. It has been shown that there are moments when there is coherence, and there are moments when there is no coherence (e.g., Mauss et al., 2005). While this is sufficient to reject the notion of specific expressions as diagnostics at a given moment (e.g., Krumhuber & Kappas, 2022), it is not sufficient to generate behavior of an artificial system in real-time, ongoing interactions. Here, it is necessary for a system to decide what behavior to show.

Having access to expressive artificial systems is a chance to test some assumptions regarding the importance of expressive behavior between humans. There is broad evidence that situational context affects the interpretation of facial and vocal behavior (e.g.,Calbi et al., 2017; Wieser & Brosch, 2012). Interestingly, recent advances in deep learning approaches, such as GPT-4, are beginning to enable machines to reason about situations in human-like ways (e.g., Tak & Gratch, 2023) which may open new windows into analyzing how interaction partners integrate situational and expressive factors to construct social meaning.

We need to have a better understanding of automatic analysis of objective behavior, as there are numerous factors relating to the quality of the recordings, as well as biases in samples, such as race or age, that affect the reliability of machine learning approaches.

There is much reason to believe that research and development in the area of artificial affect will benefit from a closer relationship between emotion researchers and engineers. However, affect is only one facet of interpersonal interaction and this requires also the integration of other areas, such as communication science, linguistics, and ethology. Robots that only embody text, as produced by some AI and flaunt emotional expressions at moments when the contents seem to have an emotional tone, or simply mimic the interactant will neither resemble real human behavior, nor will they be ultimately successful. *These would not be the droids we are looking for*. We need ethologically valid models of interaction that embed affect as one of their elements. There is much to do.

## Additional Information

### Availability of Data and Material

Data sharing not applicable to this article as no datasets were generated or analyzed during the current study.
